# A Coordinated Approach by Public Domain Bioinformatics Resources to Aid the Fight Against Alzheimer’s Disease Through Expert Curation of Key Protein Targets

**DOI:** 10.3233/JAD-200206

**Published:** 2020-09-01

**Authors:** Lionel Breuza, Cecilia N. Arighi, Ghislaine Argoud-Puy, Cristina Casals-Casas, Anne Estreicher, Maria Livia Famiglietti, George Georghiou, Arnaud Gos, Nadine Gruaz-Gumowski, Ursula Hinz, Nevila Hyka-Nouspikel, Barbara Kramarz, Ruth C. Lovering, Yvonne Lussi, Michele Magrane, Patrick Masson, Livia Perfetto, Sylvain Poux, Milagros Rodriguez-Lopez, Christian Stoeckert, Shyamala Sundaram, Li-San Wang, Elizabeth Wu, Sandra Orchard, IMEx Consortium, UniProt Consortium

**Affiliations:** a Swiss-Prot Group, SIB Swiss Institute of Bioinformatics, Centre Medical Universitaire, Geneva, Switzerland; b Protein Information Resource, Georgetown University Medical Center, Washington, DC, USA; c Protein Information Resource, University of Delaware, Newark, DE, USA; d European Molecular Biology Laboratory, European Bioinformatics Institute (EMBL-EBI), Wellcome Trust Campus, Hinxton, Cambridge, UK; e Functional Gene Annotation, Preclinical and Fundamental Science, Institute of Cardiovascular Science, University College London (UCL), London, UK; f Perelman School of Medicine, University of Pennsylvania, Philadelphia, PA, USA; g Alzforum, Cambridge, MA, USA

**Keywords:** Alzheimer’s disease, Cytoscape network analysis, data curation, database, neurobiology, protein

## Abstract

**Background:**

The analysis and interpretation of data generated from patient-derived clinical samples relies on access to high-quality bioinformatics resources. These are maintained and updated by expert curators extracting knowledge from unstructured biological data described in free-text journal articles and converting this into more structured, computationally-accessible forms. This enables analyses such as functional enrichment of sets of genes/proteins using the Gene Ontology, and makes the searching of data more productive by managing issues such as gene/protein name synonyms, identifier mapping, and data quality.

**Objective:**

To undertake a coordinated annotation update of key public-domain resources to better support Alzheimer’s disease research.

**Methods:**

We have systematically identified target proteins critical to disease process, in part by accessing informed input from the clinical research community.

**Results:**

Data from 954 papers have been added to the UniProtKB, Gene Ontology, and the International Molecular Exchange Consortium (IMEx) databases, with 299 human proteins and 279 orthologs updated in UniProtKB. 745 binary interactions were added to the IMEx human molecular interaction dataset.

**Conclusion:**

This represents a significant enhancement in the expert curated data pertinent to Alzheimer’s disease available in a number of biomedical databases. Relevant protein entries have been updated in UniProtKB and concomitantly in the Gene Ontology. Molecular interaction networks have been significantly extended in the IMEx Consortium dataset and a set of reference protein complexes created. All the resources described are open-source and freely available to the research community and we provide examples of how these data could be exploited by researchers.

## INTRODUCTION

Alzheimer’s disease (AD) is a progressive neurodegenerative disease characterized by loss of memory, inability to process new information, loss of language function, a disturbed perception of space, inability to do calculations, indifference, depression, delusions, and eventually death. Inheritable AD (familial AD) represents less than 5% of AD cases of which 10–15% have a family history of autosomal dominant inheritance; whereas the more common, sporadic, AD with complex polygenic risk inheritance accounts for more than 90% of cases [1]. Worldwide, at least 50 million people are currently believed to be living with AD or other dementias and this number could exceed 152 million by 2050 (https://www.who.int/news-room/fact-sheets/detail/dementia). The global cost of AD and dementia is estimated to be $605 billion, which is equivalent to 1% of the entire world’s gross domestic product. Globally, governments and medical charities spend millions of taxpayer and fundraiser dollars on biomedical research into this condition. It is therefore critical that the data generated by AD research is collated, organized and available in data resources and tools to increase the pace of discovery and innovation.

AD is a complex disease which needs to be studied at many levels, from the molecular mechanisms to the cellular composition and physiology of the brain [2]. Conditions such as vascular damage and neuroinflammation are also believed to play important roles in disease initiation and progression. Our current understanding of the causes, risk factors, and sub-types of these devastating conditions have been reviewed extensively elsewhere (for example, [2–4] and they are not the subject of this manuscript. However, a number of key processes known to play a role in disease etiology and progression are briefly described to showcase the representation of selected proteins in UniProtKB and demonstrate how users can access information about both physiological and pathological aspects of the molecules.

Central to AD disease pathology are two processes: the extracellular formation of senile plaques in the grey matter of the brain which are primarily composed of amyloid-β precursor protein (APP)-derived amyloid-β (Aβ) [5, 6], and intracellular accumulation of hyperphosphorylated tau/Microtubule-associated protein tau (MAPT) protein to form neurofibrillary tangles [7, 8]. Aβ oligomers are believed to contribute to cell death by interfering with neuron-to-neuron communication at synapses [9] and restricting the source of oxygen and nutrients [10], while tau tangles block the transport of nutrients and other essential molecules inside neurons [11]. Whilst the relationship between Aβ and tau in AD is not fully understood, abnormal species of tau protein are believed to spread in a ‘prion-like’ manner between cells and its uptake may be potentiated by extracellular Aβ [12, 13]. Aβ peptides can be cleared intracellularly by microglia and other cell types [14–16], by transcytosis across the blood-brain barrier [17, 18], or by Aβ degrading enzymes, such as insulin-degrading enzyme (IDE) and neprilysin (MME) [19, 20]. Tau has been shown to be degraded via the ubiquitin-proteasome system as well as the autophagy lysosome system [21]. Disorders in clearance of Aβ and tau play a key role in the development of neurodegenerative disorders such as AD while overloading of the microglial system results in chronic inflammation [22, 23]. However, evidence has been emerging that aggregation of Aβ and tau may not be the underlying causes of disease, but may be the outcome of perturbations in cellular homeostasis in the brain, occurring years to decades prior to disease onset [2, 24]. Normal brain function may be compromised by the decreased ability of the brain to metabolize glucose and aberrant lipid metabolism, such as sluggish cholesterol transport [25]. To date, over 350 human proteins have been associated with the development of AD as researchers move toward an understanding of the underlying cellular mechanisms that drive the formation of the protein aggregates and the downstream effects these have on the brain.

The analysis and interpretation of data generated from increasing large-scale examination of patient-derived clinical samples relies on access to high-quality bioinformatics resources. The scientific content of these resources is maintained and updated by professional biocurators who extract knowledge from unstructured biological data described in free-text journal articles and convert it into both more easily digestible, high-level summaries and a structured, computable form. The latter both enables large-scale data analyses, for example functional enrichment of sets of genes/proteins using the Gene Ontology (GO) [26, 27], and also helps to make the searching of data more productive by managing issues such as the problems caused by gene/protein name synonyms, identifier mapping, and minimizing the effect of poor quality, redundant, or misleading data. The work of these data resources helps researchers overcome known bottlenecks in data analysis, namely the time spent in discovering and collating required information, manually verifying it, and integrating it into analysis pipelines [28]. We here describe a coordinated approach to updating key public domain resources with the aim of supporting AD research, starting with the update of genes/proteins with a known role in AD biology. Accessing informed input from the clinical research community was an essential part of this process and was critical in defining where curation effort was focused. We also illustrate the way this coordinated update can be used by researchers to answer questions pertaining to the complex etiology of AD.

## METHODS AND MATERIALS

### Identifying disease-related proteins

A recent initiative by the UniProt Knowledgebase of protein sequences and annotations [29] to update the proteins which play a role in the initiation and development of AD, coordinated with the curation of their interactions and the complexes they form, has been funded by the NIH National Institute on Aging (NIA). At the start of this annotation project, curators were faced with two main problems—an accurate description of the various forms of AD and identification and prioritization of the proteins associated with the disease. AD is generally classified into early and late-onset forms, with genetic variants or risk alleles [30] associated with each condition providing a further sub-classification. In order to identify key AD-related proteins appropriate for update and reannotation, UniProt curators reached out to members of the AD clinical and research communities, leveraging contacts made through the NIH NIA programs and a collaboration with the Alzheimer’s Research UK (ARUK) funded GO project at University College London (UCL) [31, 32]. Workshops were organized to help database providers understand how their resources are used by the research community, and conversely for the research community to directly input into the curation process. Attendees were asked to identify proteins which played a key role in the disease, or which had been associated with disease even if a clear molecular mechanism explaining this association had yet to be identified. Additional candidates were provided by Alzforum (https://www.alzforum.org), the Agora portal (https://agora.ampadportal.org), collected from targeted research groups, and from literature searching. The main pathway resource consulted was WikiPathways which provided an overview of the disease process (https://www.wikipathways.org/index.php/Pathway:WP2059). Drug target resources included the ChEMBL database [33] and the OpenTargets platform [34], taking only high scoring (0.8 to 1) targets associated with AD from the latter. To build the AD-centric protein-protein interaction network, data was downloaded from the IntAct molecular interaction database [35], limited to interactors with an MIscore of >0.45 (see explanation below). Proteins were prioritized for curation following a ranking system, i.e., 1) proteins known to play a functional role in AD pathways and known drug targets for AD, 2) proteins known to have an association to AD, e.g., through a genome wide association study (GWAS) study but for which a molecular mechanism has yet to be identified, and 3) proteins that physically interact with those defined in (1) or (2). A copy of this list, as of UniProt release 2019_10 is available as Supplementary Table 1.

### Protein annotation

Data from selected publications were transferred into the UniProtKB, GO, IntAct molecular interaction, and the Complex Portal databases, as appropriate, as previously described [26, 32, 35–37].

### Producing an AD-centric molecular interaction network

Seed proteins were identified by searching the UniProt website (Release 2019_08) for reviewed entries containing the keyword ‘Alzheimer disease’. (keyword: “Alzheimer disease [KW-0026]” AND reviewed: yes). As this keyword is only added to human entries, there was no need to further restrict the search by species. The final list is available in Supplementary Table 2.

Interactors of this list of proteins were obtained from IntAct using the PSICQUIC client app in Cytoscape Version: 3.7.1 [38]. To return an isoform- and post-processed chain-specific network the following query was used: (id:P37840* OR id:P49810* OR id:P49768* OR id:O14672* OR id:P03886* OR id:Q8IZY2* OR id:Q16643* OR id:P02649* OR id:P05067* OR id:Q92673* OR id:P03891* OR id:O95185*) AND annot: “imex curation”.

This gave a raw network containing 1,461 nodes and 2,671 edges.

The network was then filtered to: a) remove non-human interactors; b) remove duplicated interactions; c) select interactions having MIscore > 0.45.

A MIscore of >0.45 can only be achieved by interacting pairs having at least a single interaction evidence showing that the two molecules directly interact or two or more evidences of a physical interaction. The filtered isoform- and post-processed chain-specific network contains 152 nodes and 277 edges.

To enable users to access a detailed view of this network, a copy has been deposited at the NDEx data repository (https://www.ndexbio.org/#/network/49e43d68-939b-11ea-aaef-0ac135e8bacf) [39]. Users may alternatively download an updated set of the data used to derive an AD-focused interaction network by pasting the query annot: “dataset:Alzheimers” into the IntAct website (https://www.ebi.ac.uk/intact). Using the Advanced Search capabilities will enable further filtering of the results of this query.

To perform the ClueGO functional enrichment analysis, all isoforms and post-processed chains were collapsed to the canonical identifiers in UniProtKB, all leaves (proteins not directly connected in the network) were removed and the complexes were demerged into protein subunits. The Cytoscape APP ClueGO version 2.5.0 [40] was then used, implementing the following parameters:

1 Organism analyzed: Homo Sapiens [9606].
2 Identifier types used: UniProtKB
3 #Genes in custom reference set: 3001 human proteins extracted from UniProt having tissue-specificity=’brain’
4 Ontology used: GO_BiologicalProcess-EBI-QuickGO-GOA_20.11.2017_00h00 and REACTOME_Reactions_20.11.2017
5 Evidence codes used: All
6 Statistical Test Used = Enrichment (Right-sided hypergeometric test), Correction Method Used = Bonferroni step down
7 Min GO Level = 8
8 Max GO Level = 20
9 GO Fusion = true
10 GO Group = true
11 Kappa Score Threshold = 0.4
12 Over View Term = SmallestPValue
13 Group By Kappa Statistics = true
14 Initial Group Size = 1
15 Sharing Group Percentage = 60.0

## RESULTS

All known human protein-coding genes have been curated by experts within the UniProtKB database (https://www.uniprot.org) with, as far as possible, all the protein products encoded by one gene described in a single reviewed entry [29, 36]. Each entry groups all the protein isoforms expressed by that gene, with positional features such as binding domains, post-translational modifications and amino acid variants mapped to a representative sequence. Isoforms yet to be integrated are maintained in unreviewed entries but are accessible as part of the complete human proteome reference set (UniProt Proteome UP000005640) and can also be viewed in the corresponding reviewed entry on the website as a result of an automatic gene-centric mapping. Expert curators summarize knowledge extracted from biomedical literature in sections describing different aspects of protein biology relevant to those gene products, these can include function, enzymatic activity, subcellular location, and links to disease conditions. For example, over the period of this annotation project PSEN1 (UniProtKB P49768) had data from 43 publications added to its entry in UniProtKB, enhancing the ‘Function’ section, and including details of the functional roles played by specific domains within the protein. Information on disease linked variants and the effects of point mutations on protein behavior were also added.

Proteins do not operate in isolation and details of their interactions with other molecules are manually curated by the IMEx Consortium of interaction databases (https://www.imexconsortium.org) [41] via the IntAct database [35], from where a filtered subset of high confidence binary protein-protein interactions is imported back into the ‘Interaction’ section of the corresponding UniProtKB entries. Proteins also form higher-order, functional assemblies and descriptions of stable protein complexes are curated into the Complex Portal (https://www.ebi.ac.uk/complexportal), giving details of complex content, stoichiometry, and topology in addition to function and 3D structure, when available [37]. Again, these data can be accessed from the appropriate UniProtKB records. In parallel, biocurators link these proteins and protein complexes to appropriate GO terms describing their biological function, the cellular processes in which they play a role, and the cellular compartment in which they are found. The GO is a biomedical ontology which describes these aspects of protein behavior in a consistent and computer-accessible manner [26, 27]. Linking gene products to GO terms means that researchers can use the resulting annotations to interpret high-throughput datasets using GO term enrichment.

The NIA-funded annotation project resulted in data from 954 papers being added to the UniProtKB, GO, and IMEx databases, with 299 human proteins and 279 orthologs updated in UniProtKB. 7,045 binary interactions were added to the IMEx human molecular interaction dataset.

### Understanding the function of AD-associated proteins

**Fig.1:**
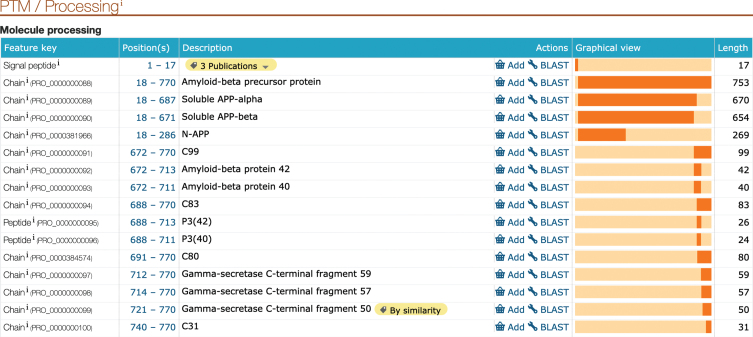
Screenshot showing the UniProtKB description of the products of amyloid-beta precursor protein post-transcriptional modifications and processing. This information is available in the UniProtKB P05067 entry for amyloid-beta precursor protein (APP).

UniProt curators provide high-quality literature sourced annotations for experimentally characterized proteins across diverse protein families. These data are presented both in free text fields and in structured mappings to the underlying protein sequence to enable users to understand how, for example, a post-translational modification to a specific residue can drive a change in protein behavior. The proteins identified by AD domain experts were subjected to an intense literature review and corresponding update of the relevant annotation fields in order to help researchers understand both the physiological role these entities play in a cell, and how this relates to the pathological disease condition. As described above, this includes a full review of both protein isoforms and protein chains formed by post-translational processing of the full-length gene product. This is particularly important in the case of AD-related proteins as amyloid plaque formation is a consequence of disregulated protein cleavage [42]. APP (UniProtKB P05067) is a ubiquitously expressed type I transmembrane protein which functions as a cell surface receptor with roles in neurite growth, neuronal adhesion, and axonogenesis. The protein consists of a large ectodomain, a single membrane spanning domain and a short cytoplasmic tail. The ectodomain comprises two highly conserved E1 and E2 domains, involved in metal (copper and zinc) and heparin binding. APP undergoes extensive post-translational modification and proteolytic processing to generate peptide fragments. The cleavage products of APP are all described at the residue level in the UniProtKB database, with stable identifiers allowing unambiguous recognition of each proteoform when described (Fig. 1).

As detailed in the appropriate UniProtKB records, APP processing is initiated either by *α*-secretase/ADAM10 (UniProtKB O14672) cleavage within the Aβ region, or by β-secretase (BACE1/2, UniProtKB P56817/Q9Y5Z0) cleavage at the N-terminus of Aβ, leading to the secretion of large soluble ectodomains, termed soluble APP*α* (APPs*α*, UniProtKB PRO_0000000089) and soluble APPβ (APPsβ, UniProtKB PRO_0000000090), respectively. Subsequent processing of the C-terminal fragments by the *γ*-secretase complex (Complex Portal:CPX-2176/CPX-4231/CPX-4232/CPX-4233), as well as processing along non-canonical pathways, result in numerous fragments, which have different and partially opposite functional properties. During amyloidogenic processing, APP is sequentially cleaved by β- and *γ*-secretases to mainly generate Aβ_40_ (UniProtKB PRO_0000000093), and Aβ_42_ (UniProtKB PRO_0000000092) fragments.

Many of the AD-associated proteins prioritized for update (Supplementary Table 1) are enzymes, which may be responsible for the proteolytic processing of longer protein chains as described above, catalysis of metabolic reactions, or generation/removal of post-translational modification sites. Enzymatic function is now described in UniProtKB using Rhea (https://www.rhea-db.org), a comprehensive and non-redundant resource of expert-curated biochemical reactions [43], as a vocabulary to annotate and represent enzyme-catalyzed reactions. Rhea uses the ChEBI (Chemical Entities of Biological Interest) ontology to describe reaction participants, their chemical structures, and chemical transformations [44]. Additional small molecule interactions, such as cofactor binding sites are also described within UniProtKB using ChEBI. Sophisticated searches within UniProtKB now allow the researcher to identify metabolic networks and predict new pathways for drug production. For example, alterations in sphingolipid metabolism have been detected in AD, with levels of SPHK1 (UniProtKB Q9NYA1) downregulated and, conversely, levels of SPHK2 (UniProtKB Q9NRA0) upregulated [45]. Both entries for these proteins have been updated in UniProtKB, where it is now possible to visualize the chemical reaction, balanced for mass and charge (at an arbitrary pH of 7.3) as described by Rhea, and cofactors linked to the corresponding entry in ChEBI (Fig. 3A).

**Fig.2:**
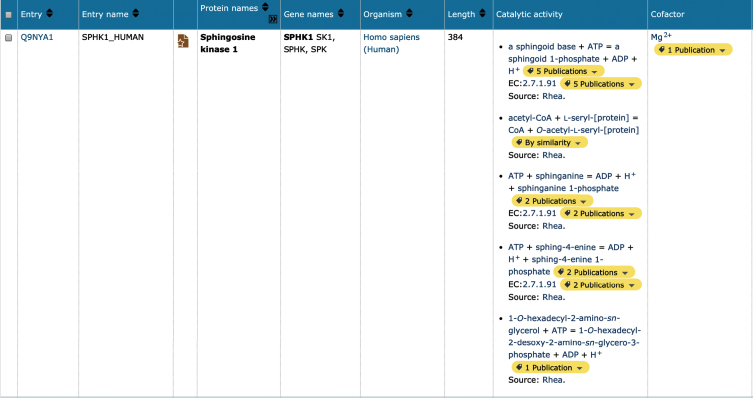
Screenshot showing the results of a UniProtKB search for human Sphingosine kinase 1. The UniProtKB database was queried for the term ‘SPHK1’. The top hit (human) in the results table is displayed. It is possible to customize this view to select additional data fields from the UniProt record, in this case the column options ‘cofactor’ and ‘catalytic activity’ were added to the results table.

**Fig.3:**
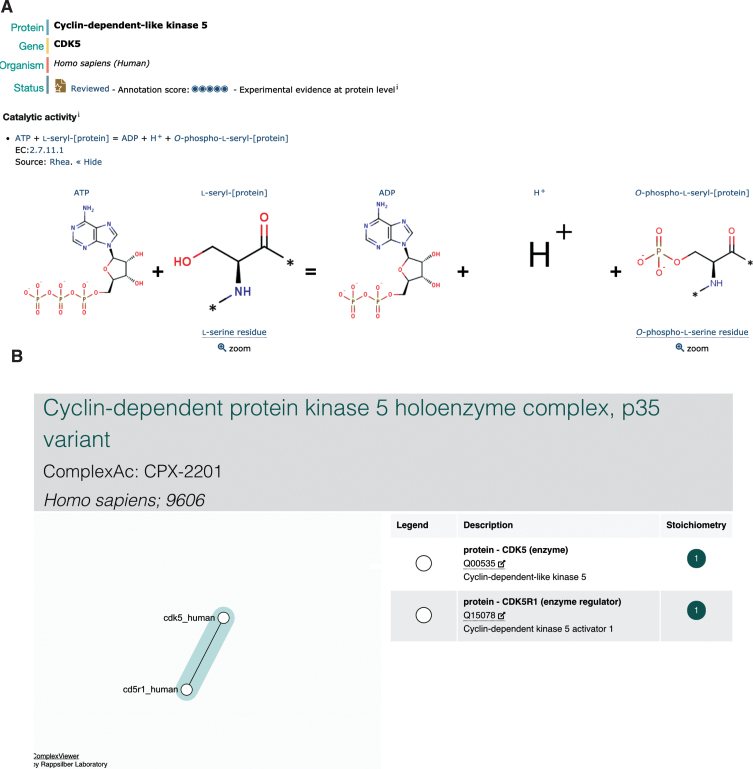
Representation of CDK5 in UniProtKB and the Complex Portal. A) The representation of CDK5 catalytic activity by Rhea within the UniProtKB entry. B) The Complex Portal display of the CDK5-p35/CDK5R1 complex which can be found by searching for either of the proteins, or by complex name.

Tau/MAPT (UniProtKB P10636) is a microtubule-associated protein predominantly expressed in the axons of neurons [46]. Tau is a naturally unfolded protein with an extended structure; however, in AD brains, tau is accumulated in a hyperphosphorylated state in a unique filamentous structure with paired helical filaments of 10 nm diameter with 80 nm periodicity [47]. The phosphorylation of tau regulates both its functional ability to assemble and stabilize microtubules and also its pathological structure [48], and the 441 amino acid isoform of tau (UniProtKB P10636-8) has 45 serine, 35 threonine, and 5 tyrosine residues, resulting in a total of 85 potential phosphorylation sites [49]. CDK5 (UniProtKB Q00535) is one enzyme known to play a role in the phosphorylation of tau [50], priming tau for further phosphorylation events by the hierarchical kinase GSK3B (UniProtKB P49841) by modifying an upstream +4 (or +3) site, (S/T)xx(x)p(S/T). Again, this chemical reaction has been updated in UniProtKB (Fig. 3B), where it is also possible to identify the resulting phosphorylated residues in the corresponding entry for tau. CDK5 is activated by p35/CDK5R1 (UniProtKB Q15078), the resulting complex (Complex Portal:CPX-2201) then being recruited to membranes via the N-terminal p35 myristoylation site [51]. p35/CDK5R1 is a protein with a short-life span which is cleaved by calpain (Complex Portal:CPX-2674/CPX-4302) into a p25 C-terminal fragment (UniProtKB PRO_0000004795) when neurons suffer from stress or encounter death signals. p25/CDK5R1 has a longer half-life and this complex (Complex Portal:CPX-3142) dissociates from the plasma membrane into the nucleus, where it can phosphorylate additional proteins [52].

### Linking amino acid variation to functional consequence

AD-causing mutations in APP (UniProtKB P05067), PSEN1 (UniProtKB P49768), and PSEN2 (UniProtKB P49810) affect the generation of Aβ peptides, changing the relative ratio of Aβ_42_ to Aβ_40_ peptide [53]. The longer Aβ_42_ peptides seem to be more prone to aggregation, and increased ratios of Aβ_42_/Aβ_40_ are thought to play a role in AD pathogenesis. It is therefore important to document all APP, PSEN1, and PSEN2 variants that lead to a change in this ratio. About 1% of AD cases develop as a result of mutations within APP or the genes encoding the PSEN1 and PSEN2 proteins present in the *γ*-secretase complex; however, those inheriting a known AD-associated APP or PSEN1 variant will develop the disease, whereas a slightly lower risk (95%) is associated with inheriting a known AD variant in PSEN2 [54]. Individuals with AD mutations in any of these three genes tend to develop early-onset disease, with symptoms developing before the age of 65, sometimes as early as age 30. Understanding how a genetic variation changes protein function or expression levels is essential for our understanding of genetic disease and the ability to identify those variants which are causal. UniProtKB curators capture nonsynonymous variants described in the literature with, when available, detail on the phenotypic or pathogenic consequences on the amino acid change. UniProt also receives input (publications and suggested annotations) from expert groups, e.g., Alzforum, who collects detailed variant information about AD proteins from the literature. To date, UniProtKB records contain information on over 30,000 variants linked to Mendelian diseases in more than 13,000 human protein sequence records [55] and work is ongoing to standardize variant interpretations through the incorporation of American College of Medical Genetics and Genomics (ACMG) guidelines and the ClinGen pathogenicity calculator into the curation workflow. Cross-references to variant resources such as dbSNP (https://www.ncbi.nlm.nih.gov/snp/) and Ensembl (https://www.ensembl.org), and disease-specific databases such as NIAGADs (https://www.niagads.org/) are added. Additional variant data is imported from large-scale studies such as 1000 Genomes and ExAC, and again mapped to the protein sequence and made available via the Proteins API (https://www.ebi.ac.uk/proteins/api/doc/).

**Fig.4:**
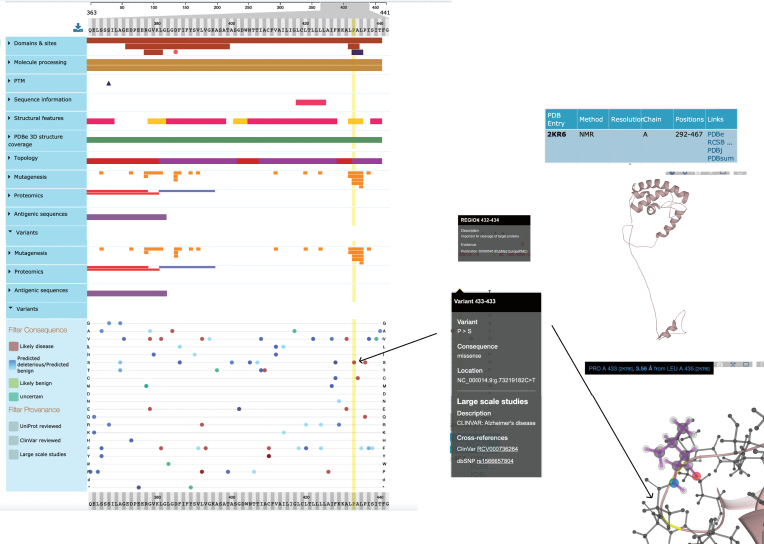
Simplified view of ProtVista for Human PSEN1 (UniProtKB P49768). To investigate the effect of a specific variant (p.Pro433Ser) of human PSEN1 protein, the user can look at its potential effect on active sites and domains. Clicking on the variant at position 433 shows it to be positioned in the PAL domain, required for normal active site conformation and also in a region important for cleavage of this protein. The position of this variant is also highlighted in the NMR structure of this protein.

UniProtKB acts as an integrative layer, enabling users to align genomic variants with enzyme active sites, modified residues, the phenotypic consequence of site-directed mutagenesis and binding domains mapped to the residue level. An exact mapping of the Ensembl translation to a UniProtKB sequence enables the calculation of UniProtKB positional annotations to their genomic coordinates and these mappings are continually reviewed and updated by both UniProt and Ensembl curation teams [56]. Thirty-four different positional annotation types are currently aligned with the genome sequence. An additional 17,371 mutations which map to the genome have been supplied by the IMEx Consortium which captures the effects of point mutations on molecular interactions, using controlled vocabulary terms to describe whether these increase, disrupt, or cause an interaction to occur [57]. Again, these site-directed mutations have been mapped to the underlying UniProtKB protein sequence and can be used to understand the effect a genomic variant may have on a local protein interaction network. Further to this, in collaboration with PDBe through the Structure Integration with Function, Taxonomy, and Sequences resource (SIFTS; https://pdbe.org/sifts/), UniProtKB maps between protein structure and protein sequence, so that a knowledge of protein conformation can contribute to an understanding of protein function [58]. These data are all displayed in UniProtKB using the ProtVista visualization tool [59] which allows the graphical alignment of sequence feature data to the linear protein sequence and from there to the 3D structure (Fig. 4).

Late-onset AD is observed in >90% of patients, and the APOE (UniProtKB P02649) allele E4 is strongly associated with these cases. APOE is a plasma lipoprotein which transports lipids between cells and tissues. Abnormal cholesterol metabolism associated with allele E4 is believed to mediate cell type-specific AD pathology, including Aβ upregulation and impaired synaptic function in neurons, reduced synapse elimination activity in astrocytes, impaired remyelination in oligodendrocytes, and Aβ accumulation and inflammatory response in microglia [60]. The most common allele in the human population, and that present on the reference genome GRCh38, APOE*3 is the displayed sequence in the UniProtKB entry, with all three possible alleles fully described in the Polymorphism section of the entry. Sequence variants, single amino acid polymorphisms, and other sequence annotations, have then been described relative to that allele with the alignment of the APOE sequence to the reference genome then allowing the integration of genomic and protein data. In the recent curation project, APOE had information from 40 new references added to the entry.

### Enabling functional Insights Into large-scale AD datasets through network analysis

AD is not a single disease but a number of separately-triggered conditions [61] which share the same pathological phenotype, suggesting that these conditions may have many downstream processes in common. Understanding how proteins associated with AD are linked in the interacting network of molecules that drive cellular processes may help to identify proteins which are critical for initiating or driving the disease condition as potential therapeutic targets. Network-based analysis is a powerful technique for extracting biological insights from large datasets, enabling researchers to identify clusters of interacting molecules which participate in the same biological process or are members of the same physical complex. Protein interaction networks can help researchers understand the interconnectivity of both intra- and extracellular signaling, while studying network topology can give information about biological function and properties of the component molecules. Merging external ‘omics data, such as transcriptomics, proteomics, and genome-wide association (GWA) studies, with the network can indicate tightly associated nodes of co-regulated proteins. An understanding of the processes associated with these networks can be further investigated by using GO annotations or Complex Portal data.

The IMEx Consortium curates to a detailed curation model, i.e., all aspects of an interaction experiment, including host organism, interaction detection, and participant identification methodologies and full details of the constructs, including binding domains and the effects of site-directed mutations, are captured [41, 57]. All this information is accurately mapped to controlled vocabulary terms, in particular those described by the HUPO PSI-MI CV. Interactions are not limited to protein-protein but increasingly also include protein-small molecule, protein-protein complex, protein-ncRNA, and protein-gene interactions using identifiers from ChEBI, Complex Portal, RNACentral (https://www.rnacentral.org), and Ensembl, respectively, to identify the respective entities. This enables the IMEx databases to fully capture the differences in interacting molecules observed with different APP isoforms (UniProtKB P05067-4/P05067-8 IntAct:EBI-21132406/EBI-21132308) [62] or by monomeric (UniProtKB PRO_0000000092) versus oligomeric (Complex Portal CPX-1134) Aβ_42_ (IntAct:EBI-20818781/EBI-20821761) [63]. The effects of mutagens, site directed to mimic known variants can also be described, for example the interactome of MAPT/Tau (UniProtKB P10636) p.Pro618Leu variant (dbSNP:rs63751273) with a known link to frontotemporal dementia [64], which reduces the ability of MAPT/Tau to promote microtubule assembly and accelerates aggregation of tau into filaments has been compared to that of the wild-type protein (IntAct:EBI-20800792/EBI-20799058) [65]. Data on the effect of site-directed mutations on molecular interactions is available as a downloadable file from the IntAct website (ftp://ftp.ebi.ac.uk/pub/databases/intact/current/various/mutations.tsv) and is also exported to the UniProtKB ProtVista viewer to provide additional understanding of how a particular amino acid variant may affect protein function.

**Fig.5:**
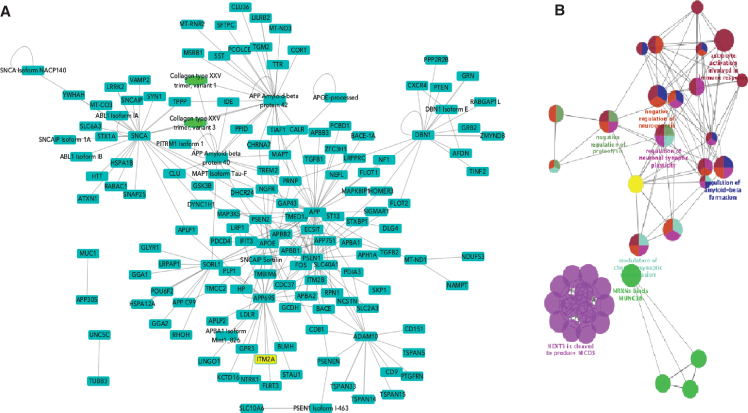
Networks built from Alzheimer’s disease relevant proteins. A) High-confidence network with isoforms and post-processed chains acting as distinct nodes. Seed proteins are those to which the Alzheimer Disease keyword has been added in UniProtKB. Blue squares represent proteins, green ovals represent protein complexes. Nodes have been collapsed to canonical sequence/gene level. B) ClueGO functional enrichment analysis of network shown in B.

Any protein interaction network built using current data will at best be partial, as we are far from having achieved full coverage of the human interactome. However, a more immediate concern is the quality of the networks being used for analysis, which are currently often created by combining data from many resources with little attention to the source(s) of the binary interactions and the methodology by which they were generated. The detailed curation model of the IMEx curation enables data filtering on many levels and thus enables the building of high-quality networks. The addition of AD-relevant protein interactions as a part of the curation marathon described above has enriched the interactome of AD-related proteins by several thousand binary interactions and is a significant addition to previous work by the IMEx curators in building the APP interactome [66]. To demonstrate the utility of these data for AD researchers, high confidence interaction networks could be built using both protein interactors described at the isoform/post-processed chain level and also following the collapse of this level of detail to the consensus sequence selected (Fig. 5A). In both cases, the seed proteins were those to which the Alzheimer Disease keyword has been added in UniProtKB. The raw network contained 1,461 nodes and 2,671 edges. This was then filtered by MI score > 0.45 [67] to produce a high-confidence sub-network, restricted to human-only interactions and redundant interaction evidences were merged, reducing this to 152 nodes and 277 edges. Remapping isoforms and post-processed chains to the canonical sequence level further reduced this to 136 nodes and 179 edges. This final network was analyzed using ClueGO, a Cytoscape App that visualizes non-redundant biological GO terms for large clusters of genes in a functionally grouped network (Fig. 5B). In this case, the network was filtered for ‘Biological Process’ term enrichment. Terms such as ‘regulation of amyloid-beta formation’, ‘regulation of synaptic plasticity’, ‘astrocyte activation’ (linked to AD pathology [68]), and child terms of Notch1 signaling (known to be altered in AD [69]) were overexpressed in comparison to a full list of human brain proteins, suggesting that this is a biologically relevant network. This network, and subsequent ongoing expansions to the dataset, is now freely available to the research community to enable network analysis of generated data and can easily be extended to encompass, for example, all the proteins known to be expressed in the human brain by performing the relevant queries on the IntAct website. This resource will facilitate interrogation of large-scale GWAS, transcriptome and proteomics clinical datasets, and allow users to explore novel biology and enhance our understanding of the disease process [70].

The Reactome database of curated biological pathways provides a tool for visualizing user-supplied expression data as an overlay on manually curated pathway diagrams [71]. Pathways are authored by biologists who are recruited for their expertise in the area, in this case biocurators involved with the curation of AD-associated papers in UniProtKB. As a result of this curation marathon, a number of AD-related pathways are in the process of being created and will be available to researchers as another tool enabling large-scale ‘omics analysis. Reactome pathways can be further extended by adding IMEx quality filtered protein interactions to extend out the networks and these additional molecules can be included in subsequent representation analysis, a statistical (hypergeometric distribution) test that determines whether certain Reactome pathways are over-represented (enriched) in any submitted dataset.

### Further enhancing the Gene Ontology to improve interpretation of AD data

UniProtKB biocurators are the single largest contributing group to GO manual annotations, both as a whole but in particular for the annotation of human proteins. The recent focus on AD proteins has added to work by the UCL Functional Gene Annotation group, funded by ARUK, to associate GO terms to proteins, protein complexes, and microRNAs relevant to processes involving amyloid-beta and tau, concomitantly creating many new GO terms in the process to further enrich those branches of the ontology relevant to neuronal biology. As a proof-of-concept of the benefit of a focused annotation effort, a functional analysis was performed by Kramarz et al. in November 2018 [31] on a hippocampal proteomic dataset, identifying proteins that were differentially expressed in AD versus age-matched controls. Analyzing the data against the GO in 2018 versus an earlier version archived in 2016 showed an almost doubling of enriched GO terms and highlighted new processes with a potential role in AD, for example 23% of dysregulated hippocampal proteins now showed a contribution to a heightened immune response. The work on curating proteins and protein complexes to GO terms is being continued by the UniProt, Complex Portal, and UCL annotation teams, while the UCL team are additionally contributing GO annotation of microRNAs regulating the expression of microglial AD relevant proteins [32].

One advantage of the improved GO representation of processes related to AD, is that it can be used as a tool to search for lists of proteins relevant to a particular aspect of the disease. It is now widely acknowledged that neuroinflammation plays a key role in the pathogenesis of AD, for example through the elevation of amyloidogenesis. The list of proteins involved in any inflammatory response is long, but searching the UniProt or QuickGO (https://www.ebi.ac.uk/QuickGO) websites for proteins annotated to the GO term “neuroinflammatory response” (GO:0150076) and limiting the search to human proteins, retrieves a list of 42 reviewed protein entries (GOA release 2020-04-22), which may be connected to the disease process. The protein list can be downloaded from the QuickGO website in CSV format, along with all the GO annotations and publications from which the evidence was extracted.

## DISCUSSION

AD is a progressive brain disorder that damages and destroys brain cells, leading to loss of memory, disregulated brain function, and eventually death. In addition to the profound human suffering caused by the condition, AD and other dementias are creating an enormous pressure on both health care systems and national budgets. To understand the molecular mechanisms both triggering and subsequently driving the development of AD, researchers have designed numerous high-throughput transcriptomic, proteomic, metabolomic, and GWA studies generating vast amounts of data. The subsequent analyses and interpretation of the results from such experiments is completely dependent on functional annotation data provided by bioinformatic resources. Resources such as UniProt, the GO, and the IMEx molecular interaction networks enable researchers to take lists of genes/proteins identified in large-scale ‘Omics experiments and, for example, find clusters of co-regulated genes which may represent processes or protein complex members involved in a particular process or pathway.

The content of these core data resources is dependent on the work of skilled biocurators, reading and evaluating the scientific literature and transferring key facts to the appropriate entries. Expert manual curation is undeniably expensive, but is essential to make this information readily available to the researcher, the clinician, and to the computational biologist. By working collaboratively, contributing data to multiple specialist resources and working together to develop shared curation tools [72], the biocuration community is taking a lead in giving funders the best possible return on their investment [28]. The AD focused biocuration project described here has benefitted from governmental funding, charitable funding, from pharmaceutical company funding through a public-private partnership [32, 34] and also from previously funded work into other neurological conditions [73, 74]. While in this case the shared funding pool was serendipitous, it suggests that actively managed collaborations between funding bodies could be at least equally successful in increasing both the quantity and quality of information freely available in biomedical databases. As a result of these efforts, researchers can now access 299 disease-relevant human protein records updated in UniProtKB (as of release 2019_10), with experimental GO annotation also added, where possible. An additional 7,045 binary molecular interactions have been added to the IMEx dataset, significantly increasing the abilities of researchers to perform network analysis on large-scale datasets.

Once the data is in these resources, it is also the responsibility of database managers to ensure that users can find and access it as easily as possible. The UniProt Consortium is already working to release a disease-specific entry point to those proteins of interest which will enable researchers to navigate the network of molecules that play a role in this condition and easily find information on the function of each. An AD portal will be the first of these released. The data is also being made available through other public domain biomedical resources such as the Open Targets platform (https://www.opentargets.org) [34] which integrates evidence from genetics, genomics, transcriptomics, drugs, animal models, and scientific literature to score and rank target-disease associations for drug target identification. The UniProt Consortium is also looking to improve the ability of both scientists and clinicians to navigate from genomic disease variant to amino acid polymorphism to effect of protein structure and/or function with both graphical visualization and computational access readily available. Variant data will become more structured, thus making it more computationally accessible [55]. The value of metabolomics data derived from AD-patients will be significantly enhanced by the work on enhancing the content of Rhea and ChEBI, and ensuring that appropriate data are incorporated into UniProt and improved and updated protein sequences will increase the number of identifications made by mass spectrometry-based proteomics groups.

In conclusion, the work described above represents a significant increase in the content of a number of public domain resources specifically focused on the molecules which play a key role in AD. Many of these proteins also play a role in other neurological disorders and are, of course, of fundamental importance to the normal physiology of the brain. These ongoing and future data updates will help clinical researchers to provide insights into the molecular mechanisms underlying the development of dementia and enable more in-depth analysis of ‘Omics’-level datasets, thus supporting the development of novel treatments and tools for early diagnosis.

## DATA AVAILABILITY

UniProtKB records in which disease is caused by mutations affecting the gene represented in that entry can be found by searching https://www.uniprot.org with the term “keyword:”Alzheimer disease [KW-0026]”. An introduction to the QuickGO Gene Ontology browser can be found at https://www.ebi.ac.uk/training/online/course/goa-and-quickgo-quick-tour. Tutorials on how to search UniProt and use the tools made available by this resource and how to access data pertaining to AD in the GO are available [75, 76]. Data required to create AD-focused molecular interaction network can be obtained by pasting the query annot: “dataset:Alzheimers” into the IntAct website (https://www.ebi.ac.uk/intact) with further details on how to use this resource available at https://www.ebi.ac.uk/training/online/course/intact-molecular-interactions-ebi. Extensive tutorial materials on Cytoscape network building and analysis are available at https://github.com/cytoscape/cytoscape-tutorials/wiki, the use of ClueGO is specifically described by Bindea et al. [40, 77]. How to use the Complex Portal is described by Meldal et al. [78].

## Supplementary Material

Supplementary Material
