# Supplementary material for: A Coordinated Approach by Public Domain Bioinformatics Resources to Aid the Fight Against Alzheimer’s Disease Through Expert Curation of Key Protein Targets
Source: J Alzheimers Dis. 2020 Sep 1;77(1):257–73. doi: 10.3233/JAD-200206 (PMC7592670; doi:10.3233/JAD-200206)
Supplement: Supplementary Material [file jad-77-jad200206-s001.pdf]

Supplementary Material

A Coordinated Approach by Public Domain Bioinformatics Resources to Aid the Fight Against Alzheimer's Disease Through Expert Curation of Key Protein Targets

Supplementary Table 1. Protein entries updated during this re-annotation exercise. Priority score (1) proteins known to play a functional role in AD pathways and known drug targets for AD, (2) proteins known to have an association to AD, e.g., through a genome wide association study but for which a molecular mechanism has yet to be identified, and (3) proteins that physically interact with those defined in (1) or (2).

Gene Name	UniProt AC	Priority score
Proteins updated as of UniProt release 2019_10		
ABCA7	Q8IZY2	1
ABCG1	P45844	2
ABI3	Q9P2A4	2
ACE	P12821	2
ACER1	Q8TDN7	2
ACER2	Q5QJU3	2
ACER3	Q9NUN7	2
ADAM10	O14672	1
ADAM17	P78536	2
ADAM9	P12821	1
ADAMTS1	Q9UHI8	2
ADPRHL2	Q9NX46	1
AGPS	O00116	2
AIF1	P55008	2
AK4	P27144	2
ALK	Q9UM73	1
ALPK2	Q86TB3	2
AP2B1	P63010	1
APOE	P02649	1
APP	P05067	1
APPL2	Q8NEU8	2
ASAH1	Q13510	2
ASAH2	Q9NR71	2
ATP1A1	P05023	3
ATP2B1	P20020	1
ATP6V1A	P38606	1
B2M	P61769	2

BACE1	P56817	1
BACE2	Q9Y5Z0	2
BAIAP2	Q9UQB8	1
BCL11B	Q9C0K0	3
BIN1	O00499	1
BSN	Q9UPA5	1
C1QA	Q9Y5Z0	2
C1QTNF4	Q9BXJ3	1
C3	P01024	2
C5AR1	P21730	2
C9ORF72	Q96LT7	1
CAMK2A	Q9UQM7	3
CASP7	P55210	2
CASS4	Q9NQ75	2
CCDC6	Q16204	2
CCR2	P41597	2
CD200	P41217	2
CD200R1	Q8TD46	2
CD22	P20273	2
CD2AP	Q9Y5K6	2
CD33	P20138	1
CD44	P16070	1
CD47	Q08722	2
CD68	P34810	2
CD74	P04233	2
CDH22	P16070	1
CDK5	Q00535	2
CDYL	Q9Y232	1
CELF1	Q92879	1
CFH	P08603	1
CFI	P05156	1
CLEC4G	Q6UXB4	2
CLEC7A	Q9BXN2	2
CLNK	Q7Z7G1	2
CLU	P10909	1
COL25A1	Q9BXS0	2
CR1	P17927	1
CRHR1	P34998	1
CSF1	P09603	1

CSF1R	P07333	1
CST7	O76096	2
CTDP1	Q9Y5B0	2
CTSD	P07339	2
CX3CL1	P78423	2
CX3CR1	P49238	2
CXCL10	P02778	1
CYP27A1	Q02318	1
CYP3A43	Q9HB55	1
CYP46A1	Q9Y6A2	1
DBN1	Q16643	2
DLG2	Q15700	1
ECHDC3	Q96DC8	2
ELAVL4	P26378	1
EP300	Q09472	2
EPHA1	P21709	2
ERBB3	P21860	1
FBXL7	Q9UJT9	2
FCER1G	P30273	1
FCGR1A	P12314	1
FERMT2	Q96AC1	2
FRMD4A	Q9P2Q2	2
FUS	P35637	1
GABBR2	O75899	3
GABRA1	P14867	1
GABRB2	P47870	3
GALNT7	Q86SF2	2
GAS2L2	Q8NHY3	2
GBA	P04062	1
GBA2	Q9HCG7	2
GBA3	Q9H227	2
GCH1	P30793	2
GFAP	P14136	1
GGA1	Q9UJY5	2
GGA2	Q9UJY4	2
GGA3	Q9NZ52	2
GJA1	P17302	1
GLIS1	Q8NBF1	2
GLIS3	Q8NEA6	2

GPRASP2	Q96D09	2
GRIN2A	Q12879	1
GRN	P28799	1
HABP4	Q5JVS0	1
HBEGF	Q99075	2
HDAC1	Q13547	1
HESX1	Q9UBX0	2
HLA-DRB5	Q30154	1
HSBP2	Q16082	1
HSD17B10	Q99714	2
HSPB1	P04792	1
IDE	P14735	2
IGFBP5	P24593	1
IL10	P22301	1
IL10RA	Q13651	1
IL1B	P01584	2
IL6	P05231	2
INPP5D	Q92835	1
IQCK	Q8N0W5	2
IRF2BPL	Q9H1B7	2
ITGAM	P11215	2
ITM2B	Q9Y287	3
ITPK1	Q13572	1
KANSL1	Q7Z3B3	2
KAT8	Q9H7Z6	2
KCNJ15	Q99712	2
KCNMB2	Q9Y691	2
LBR	Q14739	2
LDLR	P01130	2
LINGO1	Q96FE5	2
LMX1B	O60663	2
LPIN1	Q14693	1
LPL	P06858	2
LRRK2	Q5S007	1
MAF1	Q9H063	1
MAPT	P10636	1
MDH1	P40925	1
MDK	P21741	1
MEF2C	Q06413	1

MEP1B	Q16820	2
MGAT3	Q09327	2
MGST3	O14880	1
MIR142/TSPOAP1-AS1	O95153	2
MME	Q495T6	2
MMP9	P14780	2
MS4A1	P11836	2
MS4A4A	Q96JQ5	2
MS4A4E	Q96PG1	2
MS4A6A	Q9H2W1	2
MSN	P26038	1
MYD88	Q99836	2
MYRF	Q9Y2G1	3
NCK2	O43639	3
NEFL	P07196	1
NFATC4	Q14934	2
NFIC	P08651	2
NME8	Q8N427	2
NPM3	O75607	1
NR1H2	P55055	1
NR1H3	Q13133	1
NR1H4	Q96RI1	1
NRN1	Q9NPD7	1
NUPR1	O60356	1
OPRL1	P41146	2
OSBPL6	Q9BZF3	2
OSTN	P61366	2
P2RX4	Q99571	2
P2RY6	Q15077	2
PACS2	Q86VP3	3
PAFAH1B1	P43034	1
PAFAH1B2	P68402	1
PAFAH1B3	Q15102	1
PAK1	Q13153	1
PAM	P19021	1
PCDH8	O95206	2
PDCL3	Q9H2J4	2
PICALM	Q13492	1
PILRA	Q9UKJ1	3

PIN1	Q13526	2
PLCB1	Q9NQ66	1
PLCG2	P16885	1
PLD3	Q8IV08	2
PLEC	Q15149	2
PLXNA1	Q9UIW2	1
PLXNB1	O43157	1
PPARA	Q07869	1
PPARGC1A	Q9UBK2	2
PPP3CA	Q08209	1
PREPL	Q4J6C6	1
PRKCA	P17252	1
PSEN1	P49768	1
PSEN2	P49810	1
PTK2B	Q14289	1
PTN	P21246	1
PTPRC	P08575	2
PTPRG	P23470	2
RAB10	P61026	2
RAB3A	P20336	1
RABEP2	Q9H5N1	1
REST	Q13127	1
RHEB	Q15382	2
RIPK1	Q13546	2
RORA	P35398	2
RPH3A	Q9Y2J0	1
RSPO2	Q6UXX9	3
RTN4	Q9NQC3	1
RXRA	P19793	1
S1PR1	P21453	1
SAMD12	Q8N8I0	3
SCG3	Q8WXD2	1
SCIMP	Q6UWF3	2
SCN2A	Q99250	1
SEPT5	Q99719	1
SEPT8	Q92599	3
SERF1A	O75920	2
SERPINB1	P30740	2
SGPL1	O95470	1

SGPP1	Q9BX95	1
SGPP2	Q8IWX5	2
SIRPA	P78324	2
SLC10A2	Q12908	2
SLC24A4	Q8NFF2	2
SLC2A8	Q9NY64	1
SLC30A3	Q99726	1
SLC4A10	Q6U841	1
SNCA	P37840	1
SORL1	Q92673	1
SPCS1	Q9Y6A9	1
SPHK1	Q9NYA1	1
SPHK2	Q9NRA0	1
SPI1	P17947	2
SPTLC1	O15269	1
SPTLC2	O15270	1
SPTLC3	Q9NUV7	1
SPTSSA	Q969W0	1
SPTSSB	Q8NFR3	1
STX1A	Q16623	1
STXBP5L	Q9Y2K9	1
SULT2A1	Q06520	1
SYNGAP1	Q96PV0	1
SYNPO	Q8N3V7	1
SYT12	Q8IV01	1
TDP-43	Q13148	1
TGFB1	P01137	1
TGFBR2	P37173	1
TIMP3	P35625	1
TLR2	O60603	2
TLR5	O60602	1
TM2D3	Q9BRN9	2
TMEM119	Q4V9L6	2
TNFA	P01375	2
TNFRSF1A	P19438	2
TNFRSF1B	P20333	2
TNFRSF21	O75509	2
TNRC6A	Q8NDV7	3
TREM2	Q9NZC2	1

TRIP4	Q15650	2
TSPAN14	Q8NG11	2
TSPOAP1	O95153	2
TTC3	P53804	2
TYROBP	O43914	1
UFC1	Q9Y3C8	3
UGCG	Q16739	2
UNC5C	O95185	2
USP8	P40818	3
VGF	O15240	1
WDR81	Q562E7	3
WWOX, MAF	Q9NZC7	2
ZNF423	Q2M1K9	2

Supplementary Table 2. A list of the proteins used to seed the interaction networks described in Figure 5.

Entry	Entry name	Gene names
P49768	PSN1_HUMAN	PSEN1 AD3 PS1 PSNL1
P05067	A4_HUMAN	APP A4 AD1
P02649	APOE_HUMAN	APOE
O14672	ADA10_HUMAN	ADAM10 KUZ MADM
P49810	PSN2_HUMAN	PSEN2 AD4 PS2 PSNL2 STM2
Q8IZY2	ABCA7_HUMAN	ABCA7
Q92673	SORL_HUMAN	SORL1 C11orf32
O95185	UNC5C_HUMAN	UNC5C UNC5H3
Q16643	DREB_HUMAN	DBN1 D0S117E
P37840	SYUA_HUMAN	SNCA NACP PARK1
P03886	NU1M_HUMAN	MT-ND1 MTND1 NADH1 ND1
P03891	NU2M_HUMAN	MT-ND2 MTND2 NADH2 ND2
